# An exploration of influences on women’s birthplace decision-making in New Zealand: a mixed methods prospective cohort within the Evaluating Maternity Units study

**DOI:** 10.1186/1471-2393-14-210

**Published:** 2014-06-20

**Authors:** Celia Grigg, Sally K Tracy, Rea Daellenbach, Mary Kensington, Virginia Schmied

**Affiliations:** 1 Midwifery and Women’s Health Research Unit, Faculty of Nursing and Midwifery, The University of Sydney, Sydney, NSW, Australia; 2 School of Midwifery, Christchurch Polytechnic of Technology, Christchurch, New Zealand; 3 School of Nursing and Midwifery, University of Western Sydney, Sydney, NSW, Australia

**Keywords:** Decision-making, Place of birth, Primary maternity unit, Tertiary hospital, New Zealand, Birthplace, Childbirth, Safety, Medical model, Midwifery model

## Abstract

**Background:**

There is worldwide debate surrounding the safety and appropriateness of different birthplaces for well women. One of the primary objectives of the Evaluating Maternity Units prospective cohort study was to compare the clinical outcomes for well women, intending to give birth in either an obstetric-led tertiary hospital or a free-standing midwifery-led primary maternity unit. This paper addresses a secondary aim of the study – to describe and explore the influences on women’s birthplace decision-making in New Zealand, which has a publicly funded, midwifery-led continuity of care maternity system.

**Methods:**

This mixed method study utilised data from the six week postpartum survey and focus groups undertaken in the Christchurch area in New Zealand (2010–2012). Christchurch has a tertiary hospital and four primary maternity units. The survey was completed by 82% of the 702 study participants, who were well, pregnant women booked to give birth in one of these places. All women received midwifery-led continuity of care, regardless of their intended or actual birthplace.

**Results:**

Almost all the respondents perceived themselves as the main birthplace decision-makers. Accessing a ‘specialist facility’ was the most important factor for the tertiary hospital group. The primary unit group identified several factors, including ‘closeness to home’, ‘ease of access’, the ‘atmosphere’ of the unit and avoidance of ‘unnecessary intervention’ as important. Both groups believed their chosen birthplace was the right and ‘safe’ place for them. The concept of ‘safety’ was integral and based on the participants’ differing perception of safety in childbirth.

**Conclusions:**

Birthplace is a profoundly important aspect of women’s experience of childbirth. This is the first published study reporting New Zealand women’s perspectives on their birthplace decision-making. The groups’ responses expressed different ideologies about childbirth. The tertiary hospital group identified with the ‘medical model’ of birth, and the primary unit group identified with the ‘midwifery model’ of birth. Research evidence affirming the ‘clinical safety’ of primary units addresses only one aspect of the beliefs influencing women’s birthplace decision-making. In order for more women to give birth at a primary unit other aspects of women’s beliefs need addressing, and much wider socio-political change is required.

## Background

Childbirth and the culture surrounding it are powerful dimensions of human society [1,2]. Birthplace is an important component of birth, which can include physical, emotional, cultural and social aspects. Women make birthplace decisions within their socio-political and cultural context, which adds to its complexity. Negotiation of conflicting or competing aspects is sometimes required [3-5]. For most, their decisions match their beliefs and values, some of which may be deeply held [1,2,4]. Identifying some aspects of women’s decision-making and their beliefs regarding birthplace will inform care providers, policy-makers and planners and educators.

There is worldwide debate surrounding the safety and appropriateness of different types of birthplaces for well women having uncomplicated pregnancies. In the context of medical decision-making, many aspects of maternity care are characterised by inadequate evidence, in particular, the quantification of the risk of adverse outcomes associated with births in different settings. This research is part of the Australasian prospective cohort Evaluating Midwifery Units (EMU) study. Its primary focus is to compare the clinical outcomes for well (‘low risk’) women, intending to give birth in either an obstetric-led tertiary level maternity hospital (TMH) or a free-standing midwifery-led primary level maternity unit (PMU) in Australia or New Zealand. The New Zealand arm of the study addresses three aspects: women’s birthplace decision-making (this article), women’s birth and maternity care experiences, and an examination of transfers between primary units and tertiary hospitals. It is a mixed methods study (concurrent QUANTITATIVE + qualitative) utilising participants’ clinical outcome data, two comprehensive postnatal surveys at 6 weeks and 6 months, and data from eight focus groups. This article explores women’s birthplace decision-making and their beliefs regarding childbirth, to identify the reasons for their choice, the people and factors of influence and their relative importance.

### Literature

There is limited research on women’s birthplace decision-making between primary and tertiary units in Australasia. Most of the research in this area was undertaken in Western resource-rich countries [6-8], in particular the UK [9-15]. Overall, the research found that the strongest influence on women planning a tertiary hospital birth is the belief in the ‘safety’ of this type of facility because of the specialist services available [9,11,13-17]. By contrast, multiple reasons were given for primary unit-planned births, including closeness to home [8,13,15,17], atmosphere or feel of unit ( [8,14], A. Gallagher unpublished Masters thesis (2003), J. Howie unpublished Masters thesis (2007)), minimisation of intervention ( [7,16], Howie unpublished observations], natural birth ( [7,14,16], Gallagher unpublished observations), control [6-8,16], knowing the midwife [6,7,15], and a different expression of ‘safety’ ( [7], Gallagher unpublished observations). Women’s previous experience has been found to be an important aspect of birthplace decision-making by some [9,10,14,16,17], with the good reputation of a given unit also reported as influential [9,10]. Studies report that women know where they want to give birth and want to make their own decision, although they are sometimes prevented from doing so by organisational limitations or requirements; for example, not having an option, not being told of birthplace options and restrictive primary unit booking criteria [7,9,10,12,15].

Most of these studies comprise surveys [6-8,11,12,14,16], with some combining these with interviews or focus groups ( [9,10,15,17], Gallagher unpublished observations). The studies represent a range of contexts. For example, different types of maternity facilities primary, free-standing and/or alongside birth centres, with homebirth often included ( [7,11,14-16], Howie unpublished observations). All but two are compared with tertiary hospitals [6,7]. Some research addresses a theoretical choice - whether women would use a primary unit if available [6]. Birthplace decision-making is only one aspect of some studies [6,15-17]. Australian research conducted 25 years ago [16] and the New Zealand research to date is unpublished ( [17], Gallagher unpublished observations, Howie unpublished observations).

Limitations of existing research include small sample size ( [8,11], Howie unpublished observations), unidentified or low response rates [9,14-16], and limited account of methods (particularly qualitative aspects, compromising assessment of rigour and reflexivity) [10,11,15]. All of these published studies compare different care providers or models of care for the different types of facilities.

The present research contributes to the literature by exploring women’s birthplace decision-making within a context of women having the same model of midwifery-led care and caregiver regardless of planned or eventual birthplace. A mixed method approach enables consideration of the complexity surrounding birthplace decision-making. The large study sample of 702 women was enhanced by a high survey response rate (82%) from both the primary maternity unit (PMU) and tertiary maternity hospital (TMH) participants and multiple focus groups.

### Context

The New Zealand maternity system has continuity of care as a core tenet [18] resulting in women receiving continuity of care regardless of birthplace. Each woman chooses her own ‘lead maternity carer’ (LMC) who continues to provide care throughout her maternity experience. In 2010 78.2% of LMCs were midwives, 1.6% general practitioners (GP), 5.8% obstetricians and 14.4% of women had an unknown or no LMC [19]. All of the EMU study participants had a midwife lead carer. The midwife remains the primary caregiver even if complications arise, requiring obstetric consultation and a change of plan antenatally or a transfer between facilities during labour and birth. (For a comprehensive description of New Zealand’s unique maternity system see Grigg & Tracy [20]).

In New Zealand in 2010 85.4% of births occurred in a secondary or tertiary hospital, 10.8% in a primary unit (birth centre), 3.2% home birth and 0.6% at an unknown location [21]. Comparative data from Australia in 2009 shows 96.9% were hospital births, 2.2% birth centre (primary unit), 0.03% home and 0.06% ’other‘ location births [22]. A TMH has specialist obstetric, anaesthetic and paediatric staff and facilities on site and available at all times. A PMU has midwifery services on site and available at all times, but no medical staff or specialist facilities. In many areas women do not have the option of a PMU, following the centralisation of maternity hospitals which began in the 1920’s [21,23]. Despite the greater proportion of PMU births in New Zealand when compared with Australia, in both countries most women give birth in a hospital, in common with most other Western resource-rich countries. Arguably this reflects the predominance of the ‘medical’ model of childbirth, and the associated social belief that birth is only ‘safe’ in a hospital [24,25]. The contrasting models of childbirth – ‘medical’ (or technocratic) and ‘midwifery’ (or holistic) – have been previously identified [25,26]. Table 1 illustrates some of their key features. Arguably medicine, and more particularly obstetrics, currently holds the ‘authoritative knowledge’ [27] in childbirth and consequently the power to define the key concepts of ‘risk’ and ‘safety’ [24].

**Table 1:** Key features of medical and midwifery models of childbirth

**Medical/technocratic model**	**Midwifery/holistic/social model**
Doctor centred	Woman centred
Obstetrics: experts in pathology	Midwifery: experts in normal physiology
Body-mind dualism; classifying, separating	Holistic; integrating approach
Pregnancy is a medical condition, inherently pathological	Pregnancy is a normal human state, inherently healthy
Birth is only normal in retrospect and requires hospitalisation and medical supervision	Birth is normal physiological, social & cultural process with environment key
Technology dominant	Technology cautious
Risk selection is not possible, but risk is central	Risk selection is possible & appropriate
Statistical/biological approach	Individual/psycho-social approach
Biomedical focus	Psycho-social focus
Medical knowledge is privileged & exclusionary	Experiential & emotional knowledge valued
Intervention	Observation
Outcome: aims at live, healthy mother and baby.	Outcome: aims at live, healthy mother and baby and satisfaction of individual needs of mother/couple.

Sources: An interpretation based on Bryers & van Teijlingen [24], van Teijlingen [25], Rooks [28] and Davis-Floyd [26].

Safety of hospital birth for all women is not supported by evidence, even if safety is measured by physical outcomes alone [29]. There is significant recent evidence of lower maternal and neonatal morbidity rates for well women who plan to give birth in a PMU, resulting from lower rates of ‘interventions’ such as caesarean sections and forceps/ventouse assisted births, which have associated morbidities [27,29,30]. This evidence has the potential to redefine ‘safe’ birthplace decision-making for communities, caregivers and policy planners.

The aim of this study is to describe and explore the influences on women’s birthplace decision-making between primary or tertiary units in New Zealand.

## Methods

A mixed method methodology was chosen for the project, as the best way to address the complexity of issues around birthplace and optimise the opportunity the study provided to collect clinical outcome data and hear and give voice to women’s experiences and thoughts. It was grounded in Pragmatism [31-33], with a ‘concurrent quantitative (QUAN) + qualitative (qual)’ typology [34,35].

Three types of data were collected from the New Zealand EMU study participants: the core clinical outcome data collected for the prospective cohort study (QUAN), survey data (QUAN-qual) and focus group data (QUAL). The six week postpartum survey provided the primary data for the decision-making aspect of the study, supplemented by the focus group data. Quantitative data were analysed using descriptive statistics and the qualitative data were analysed using descriptive content analysis. The data from both sources were integrated at the interpretation stage and triangulated to assess congruence and complimentarity. Ethics approval was granted by the Upper South B Regional Ethics Committee (URB/09/12/063).

The New Zealand arm of the Australasian study was set in the Christchurch area, in Canterbury. Christchurch is the country’s second largest city, with 350,000 inhabitants. There is a TMH and four PMUs in the area, two of which are located semi-rurally outside the city boundaries (Lincoln and Rangiora), and the two city PMUs are part of other hospitals which do not offer other maternity services and they operate independently as if they were stand-alone units (Burwood and St George’s).

### Sample and Recruitment

The participants were well pregnant women (‘low risk’ based on information on the hospital booking form) booked into one of the participating maternity units. For the purposes of this study, ‘low risk’ was defined as not having any level two or three referral criteria as defined in the New Zealand Referral Guidelines [36]. For example, women who had had a previous caesarean section or were expecting twins were ineligible. Eligible women who registered with local midwives were invited to participate. Their clinical outcome data were available through the Midwifery and Maternity Provider Organisation (MMPO), which is owned by the New Zealand College of Midwives (NZCOM) and has the country’s only national maternity database. Ninety percent of the midwives were members of the MMPO; and 17 midwives, who were not MMPO members, offered to complete customized data forms.

Recruitment was undertaken by CG. Eligible women were sent a postal invite to join the study, with a follow-up phone call to those who did not respond. Additionally, some women were invited by their midwife. Recruitment began in March 2010, was suspended for one month after a major earthquake in September 2010, and stopped prematurely after a severe earthquake in February 2011. Following the February earthquake all the study sites were temporarily disrupted, due to damage of roads, sanitation and water services, and one of the PMUs was permanently closed due to safety concerns and the building was subsequently demolished. The births of participants were between March 2010 and August 2011. Approximately 30% of those invited joined the study. A total of 702 women joined the study (295 into TMH cohort and 407 into PMU cohort) based on their intended birthplace at the time they joined (any time before labour).

### Survey construction

The questionnaires used in the EMU study were similar to, and largely based on, previously validated questionnaires from English and Australian studies: the English Evaluation of a Community Based Caseload Midwifery programme at Guy’s and St Thomas’ Trust between 2005–2007 ( [37], J. Sandall personal communications), and the Australian randomised controlled trial of caseload midwifery for low risk women (COSMOS) [38]. Some questions were also included from previous work by a team in Melbourne [39-41]. All were designed to explore the self-reported health outcomes for women and babies and their perceptions and experiences of midwifery care. The questionnaires were contextualised for use in Australia and New Zealand, and used in the recent randomised controlled trial of caseload midwifery (M@NGO) [42] and in the current study. In New Zealand the survey was piloted on ten women, who would have been eligible for the study, prior to the commencement of study recruitment. Feedback was sought from the women and a small number of questions were subsequently modified.

The survey comprised nine pages and 51 questions, some of which had multiple sub-questions. The majority of questions were ‘closed’ (tick box or Likert scale), with 13 questions open ended and nine of those sought explanatory or descriptive detail. Questions covered several topics, including:

• women’s birthplace decision-making

• several aspects of their antenatal, labour and postnatal experiences and care

• their feelings and worries regarding labour and birth

• where their baby was born

• details of any antenatal change of plan or transfer in labour and how they felt about it

• their antenatal plans for feeding their baby

• details of feeding method(s) up to the time of completing the survey, and

• details of any health problems they or their baby experienced in the first six weeks.

Further details on the survey can be obtained from the author (CG). The survey aimed to provide a comprehensive coverage of women’s birthplace decision-making; pregnancy, labour and postnatal experience and care, and the wellbeing of themselves and their baby at six weeks postpartum. A second survey at six months postpartum asked women the same questions regarding the wellbeing of them and their baby, in order to identify longer term physical and emotional wellness, as a secondary outcome for the EMU study.

### Data collection

The six week postpartum survey was sent via post, unless participants chose to receive it online by giving their email address on the study consent form (60%).

Women were notified of the focus groups in the initial study invitation and invited to join with the six week survey. The eight groups were held in local community halls and arranged according to women’s intended birthplace type (primary or tertiary) and lasted sixty to ninety minutes. Two researchers, who were not known to the participants (RD, a sociologist, and either CG or MK, midwives), co-facilitated each group, and most groups had 4–6 participants (37 in total). The groups were based on a semi-structured format with eight broad questions used as a cue sheet to guide the discussion. A question about when women made their birthplace decision and the key issues they considered was specifically included. Half of the eight focus groups were held in late 2010 and the other half in early 2012. A planned separate group for Māori participants and facilitated by a Māori midwife did not proceed due to earthquake disruption.

Both the survey and focus groups addressed the issue of birthplace decision-making.

### Data analysis

The survey results reported here were analysed using SPSS software (Version 20) using descriptive statistics for the closed questions. The open-ended responses were analysed using inductive content analysis, with NVivo software (version 10.0). The postal surveys were manually entered onto the online format (SurveyGizmo) and the complete dataset was downloaded as an SPSS file. The relevant responses were then either analysed with SPSS (closed questions) or copied into Excel/Word files and imported into NVivo (open questions).

The focus groups were audio-recorded and independently transcribed, with the transcriptions reviewed by two researchers before analysis. The focus group data were analysed independently by the three researchers who participated in the groups. The coding and interpretation was then checked collaboratively, and found to be consistent. The qualitative data from the surveys were manually reviewed and inductively grouped and coded into categories. Pseudonyms are used for focus group (FG) quotes and the ‘study code’ identifier is used for survey (S) quotes.

## Results

The two groups were similar demographically – although the TMH survey respondents were statistically significantly more likely to have a higher income than the PMU respondents (Table 2). The PMU women tended to be younger, less well educated, lower income and more were Māori, while the TMH women tended to be better educated and older. These trends reflect national patterns [21], but differ from those reported in international literature, with women planning PMU births tending to be older, Caucasian, better educated and have higher incomes [43].

**Table 2:** Survey respondents’ demographics

**Demographic**	**PMU (%) n = 330**	**TMH (%) n = 228**	**p value (Chi-Square 95% CI)**
**Parity**			0.001
0	41.6	53.3	
1	36.7	37.0	
2-4	20.9	9.3	
≥5	0.9	0.4	
**Age**			0.083
<25	11.3	7.3	
25-29	33.2	25.6	
30-34	40.9	48.3	
35-39	12.8	15.8	
≥40	1.5	3.0	
**Ethnicity**			0.365
NZ European	76.0	78.2	
Māori	5.6	2.6	
Other	18.1	18.8	
**Partner**			0.748
Yes	91.6	91.1	
No	7.6	8.2	
**Education**			0.335
No post-school completed	20.2	15.7	
Apprenticeship, certificate	16.6	13.9	
Diploma	16.9	17.8	
Degree	46.2	52.6	
**Income**			0.001
< $25,000 pa before tax	6.1	6.2	
$25,001 – $50,000	29.1	15.0	
$50,001 - $75,000	30.4	31.0	
>NZ$75,000	34.4	47.8	

Of the 692 six week postpartum surveys sent out, 571 women responded, representing a response rate of 82% (80% PMU, 82% TMH). The survey began with six questions relating to women’s initial birthplace decision-making, asking them to identify where they originally planned to have their baby. The TMH was the original planned birthplace for 234 respondents (41%), one of the four PMUs for 332 (58%) and ‘other’ for <1% of respondents (home (3), home/TMH (1), home/PMU (1)). A small number of participants had changed their intended birthplace by the time they joined the study (4%). The results regarding intended birthplace are from the original intention given by respondents, as the questions which followed referred to women’s initial choice. Almost all of the respondents agreed that they were ‘happy with their choice’ (99.9%).

Of the eight focus groups, four were held in November 2010 and four in March 2012. The latter groups were delayed as a result of the earthquakes, consequently the women were between four and 17 months postpartum when they attended a focus group. A greater proportion of the 37 focus group participants had intended to give birth in the PMU (24 women), six of those were first time mothers and five women had given birth to their first baby at the TMH previously. Of the 13 TMH women five were first time mothers. Of the PMU women, two had unplanned home births and five gave birth at the TMH (all due to antenatal or pre-admission change of plan).

The results of four topic areas are discussed below.

1) *Reasons for birthplace choice*

This was an open-ended survey question with a 98% response rate and answers ranging from one to 500 words. The key categories were identified early in the process, giving the researchers the opportunity to record the frequency of similar coded responses [44]. The focus group participants were asked about the timing of their birthplace decision-making and the issues they considered in making their decision.

Survey responses from the two groups (PMU/TMH) as to the reasons for their birth place decisions were quite different illustrating apparent divergent beliefs about childbirth. Amongst the TMH group surveyed, 95% reported that the ‘specialist facilities’ and/or ‘staff’, ‘safety’ or ‘first baby fear’ were the only reason, or one of the reasons, for their choice. Terms such as “*just in case*”, “*if needed*”, “*if anything goes wrong*” were used frequently. Just over half gave only one reason for their choice. For example,

*“specialist services if needed - this is absolutely the only reason why i wanted to go to [TMH]”* (S, TMH 4018).

*“This is my first baby, so i felt safer having the baby at the hospital just in case something went wrong”* (S, TMH 3353).

The focus group TMH participants also focused almost exclusively on ‘safety’, actively choosing the TMH for its specialist facilities and avoidance of intrapartum transfer, however unlikely. Although the District Health Board policy requires well women to be transferred postnatally from the TMH to a PMU (or home), these women saw this as a price to be paid for a ‘safe’ birth. (Well women and babies are required to leave this particular TMH within 2–3 hours of birth and most transfer to a PMU for approximately 48 hours of postnatal hospital care.) In their view the primary units were seen as lacking facilities for safe birth, with the ‘nice’ environment or atmosphere there not an adequate incentive.

*“The most important thing for me is making sure that baby’s out safely, and if there is some issue then I’d hate to have gambled in my mind the risks of having a nice sort of birth if you like, or a more relaxed situation”* (FG, TMH, Meg).

In contrast, the women who planned to give birth in the PMU reported a diverse range of reasons, in both the survey and focus groups, and most survey respondents (80%) gave more than one reason for their decision. The survey responses showed the PMU’s ‘location’ was important for many, with its ‘closeness to home’ the most frequently mentioned reason (30%), as was ‘ease of access’ for labour and/or visitors postnatally. Liking something about the PMU itself was mentioned by 54% of this group – the ‘feel or atmosphere’ (28%), ‘the food’ (14%), and the ‘size’ or ‘kind’ (14%) of unit were important for many women. Another frequently mentioned reason was ‘avoidance of early postnatal transfer’ from the TMH (22%). For example,

*“More of a homely feel, close to home, less people around. Relaxed environment”* (S, PMU 3047).

*“water birth, it’s close to family and friends and home, it is personal and they don't spit you out in three hours, and wouldn’t have to transfer hospitals. also they have good food” (*S, PMU 4015).

*“I wanted a calm and home like environment where i did not feel influenced to have medical interventions if I did not need them”* (S, PMU 3378).

Focus group responses from PMU women also mirrored the survey responses, with several issues accounting for their birthplace decision. Some chose the PMU to avoid the TMH and its associated drugs or interventions, ‘hospital’ environment, postnatal transfer or poor care, while others chose the PMU for what it had to offer, such as its quiet or peaceful ‘non-hospital’ atmosphere, closeness to home, its small size or waterbirth facility.

For each group there were other reasons mentioned less frequently in the survey. A small proportion of the TMH group mentioned wanting the option or availability of ‘pain relief’ or ‘epidural’ (14%). Only 10% (24 women) of the TMH group mentioned not wanting to transfer in labour, with six of them saying they had transferred previously and didn’t want to do it again. Some mentioned their previous births, as either good or needing assistance. Others indicated that they had been recommended to go to the TMH by someone (13%); of those their midwife was the main one to recommend it (48%). A doctor (24%) or their partner (14%) were less likely to be mentioned. The word ‘natural’ was only used by two of these respondents (<1%), and 2% mentioned the availability of a pool for labouring or birthing.

*“Having the backup of medical staff on site. Availability of epidural if needed”* (S, TMH 3134).

*“went to* [PMU] *with first child but had to transfer to* [TMH] *by ambulance therefore didn’t want to repeat that experience”* (S, TMH 3055).

*“Midwife suggestion and her preferred choice/option”* (S, TMH 3103).

Amongst the PMU women less frequently mentioned reasons included the desire to avoid medical intervention (13%), and wanting a ‘natural’ birth (7%). Previous experience was raised by 26% of PMU respondents, with 14% having previously given birth at a PMU. Having previous postnatal transfer experience or having had a normal birth in the TMH were also mentioned as reasons to go to a PMU. Two women in this group had transferred from a PMU to the TMH in labour previously and chosen to return to the PMU for the next birth, having not been put off by the transfer experience. Amongst the 15% of those who had the PMU recommended to them, recommendations from ‘others’ were the most common (41%), followed by recommendation from their midwife (29%), friends (24%) and least frequently, from their partner (6%). The pool for labouring or birthing was raised by 12% of the PMU group. The caring, calmness, support or knowledge of the PMU staff (midwives) was mentioned by 11% of PMU respondents. Other factors raised included family history at the PMU and a sense of place or belonging to the unit itself or the local community, although these cannot be explored further here. For example,

*“nice, comfortable facility, private rooms etc. No major interventions offered etc., didn’t want that option! Close to my parents house and the main hospital [TMH] if need be. Also, I was born there :-)*” (S, PMU 3459).

*“Have heard good things about it from other people. Have heard midwives very knowledgeable and friendly”* (S, PMU 3128).

*“i had been there before and i liked the pool and i had to be transferred last pregnancy and i didn’t like that. I didn’t want to have anything done i didn’t really need. I knew if i needed eg. a c-section then i would be transferred in time”* (S, PMU *3210).*

The PMU focus group participants also talked of the ‘safety’ of the PMU option, which included safety from unnecessary intervention or the emotional safety created in the PMU, which enabled effective (consequently safer) labouring and birthing. They knew of the possibility of transfer to the TMH after admission to the PMU. Some even knew the current transfer rate of approximately 13% (personal communication).

*“What’s important… ultimately to have a nice safe baby, and if it’s safe and you have it somewhere [PMU], but if you need help and can get to [TMH] if you need to, then I think that’s the most important”* (FG, PMU Joy).

*“And like every other woman there is always an ambulance or a team close by, and I can’t help but think sometimes perhaps people end up in such emergency situations because they have had all the intervention”* (FG PMU Sue).

2) *The timing of the decision*

The focus groups revealed that most women appeared to have longstanding and deeply held beliefs regarding their preferred birthplace. The question of timing was only asked in the focus groups. In response to being asked *when* they decided where they would like to give birth, most used the word ‘always’ (or something similar) in their response. For example, from TMH women:

“*I always knew I would go there, because I’m very paranoid and anxious*” (Ana).

“*I decided before I’d ever decided, I knew in my mind that I wanted to birth there*” (Mia).

Most of the PMU women also expressed this sentiment, sometimes it referred to the PMU itself:and others referred to the type of birth:

“*I had always planned, pre-children, to birth at [PMU]*” (Bel).

“*I just knew that to me it’s giving birth and I didn’t feel that I had to be in a hospital*” (Ali).

“*I knew that I didn’t want drugs if I could avoid it and then I’d investigated that the easiest way not to have drugs was not to go to [TMH]*” (Sue).

Both groups believed that their choice was both ‘right’ for them and ‘safe’ for them and their baby.

**Figure 1:**
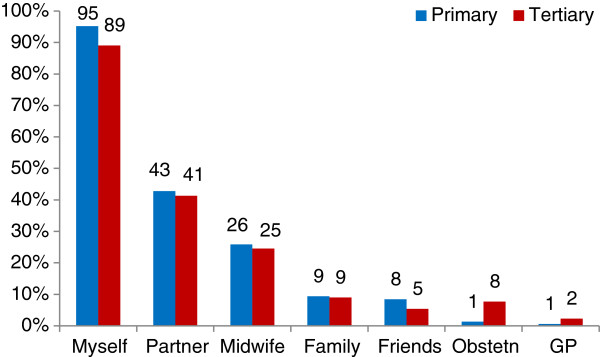
People who had ‘a lot’ of influence of women’s birthplace decision (survey).

**Figure 2:**
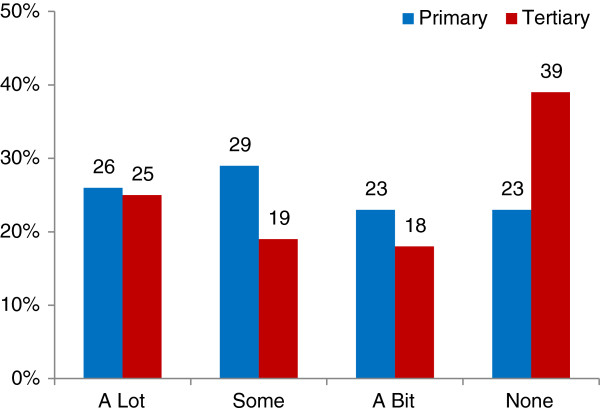
Influence of ‘my midwife’ on birthplace decision (survey).

3) *Who influenced the decision*A subsequent closed-ended question with a Likert scale asked survey participants how much the following people influenced their birthplace decision: themselves, their partner, family/whanau, midwife, doctor, obstetrician, or friend(s). The groups’ responses were very similar, with women seeing themselves as the primary decision-maker with 89% and 95% of respondents indicating that they had ‘a lot’ of influence for the TMH and PMU groups respectively (Figure 1).Women identified their husbands/partners as the second most influential people, having ‘a lot’ of influence for just over 40% for both groups (Figure 1). The women’s midwife had ‘a lot’ of influence for about 25% of both groups (Figure 1).The biggest difference in responses was in the proportion of women who said their midwife had no influence (Figure 2), with 39% of the TMH group compared with 23% of the PMU group (p < 0.001). Obstetricians had ‘a lot’ of influence on 8% and ‘none’ or ‘N/A’ influence for 90% of the TMH group, while they had ‘none’ or ‘N/A’ influence for 98% of the PMU group. Family doctors (GP) had a similar influence for both groups, having any influence at all on only 5% and 4% of respondents for the TMH and PMU groups respectively.

The focus group participants spoke of the way they chose their midwife, choosing one who supported their views of birth and birthplace plans. For example,

“*I chose my midwife based on the fact that she had a preference not to birth at [TMH]”* (Sue).

For some, the midwife was able to influence their decision, but others were not open to consider an alternative birthplace. For example,

*“my midwife tried to convince me to go elsewhere and I just wouldn’t”* (Ana).

*“my midwife said, when I met her, ‘first baby [TMH]’, it was sort of a no brainer”* (Meg).

Some changed midwives during pregnancy, or for the next pregnancy, when they perceived that they were not supported in their decision. For example,

*“I changed midwives for my next child. My second midwife was great and she just said ‘we believe in your ability to give birth however you like, we’ll have a homebirth if you want’, and so I kind of said ‘ah maybe not a homebirth!’”* (Joy).

Overall the women in the focus groups expressed confidence in their midwives, the continuity of care they provided and the maternity system, something which will be explored in another paper.

4) *What influenced the decision*

The survey used the same closed-ended format to ask the question of how much 11 factors influenced their birthplace decision on a Likert scale with ‘a lot’, ‘some’, ‘a bit’, ‘none’ and ‘N/A’ options (Table 3). The ‘none’ and ‘N/A’ responses were combined for analysis, except for the ‘my own previous birth experience(s)’ factor.

**Table 3:** The factors which might influence the birthplace decision, ‘a lot’ and ‘none’ responses

**Influencing Factor**	**PMU (%)**		**TMH (%)**		**p value**
	**A lot**	**None**	**A lot**	**None**	**(Chi-Square 95% CI)**
Closeness to home*	47	15	20	43	<0.0001
Ease of getting there*	49	10	18	40	<0.0001
Experiences of other women I heard about*	34	23	16	30	<0.0001
My own previous birth experience(s)	37	13	39	16	0.225
Availability of specialist services there*	8	67	91	2	<0.0001
The atmosphere or ‘feel’ of the unit*	55	8	12	44	<0.0001
Confidence in the hospital staff there*	39	18	59	12	<0.0001
Things I read/heard in local media	7	70	4	75	0.270
The internet*	3	87	0	95	0.006
My beliefs about labour and birth	46	22	36	27	0.105
My general or early pregnancy health*	39	27	14	56	<0.0001

*Statistically different.

**Figure 3:**
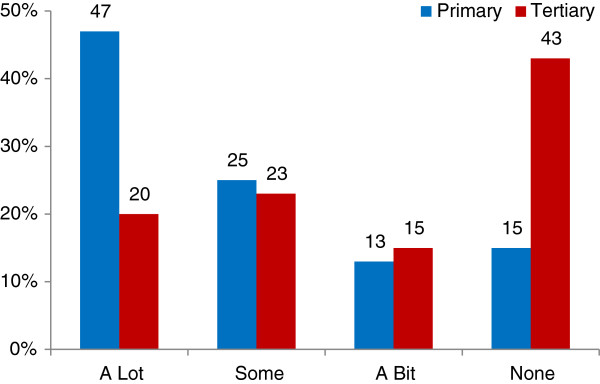
Influence of ‘closeness to my home’ (survey).

**Figure 4:**
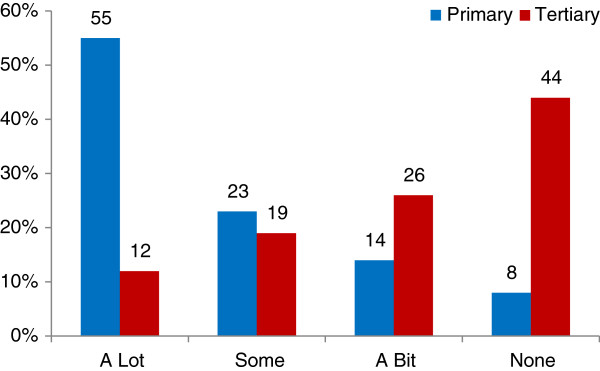
Influence of ‘atmosphere or feel of unit’ (survey).

**Figure 5:**
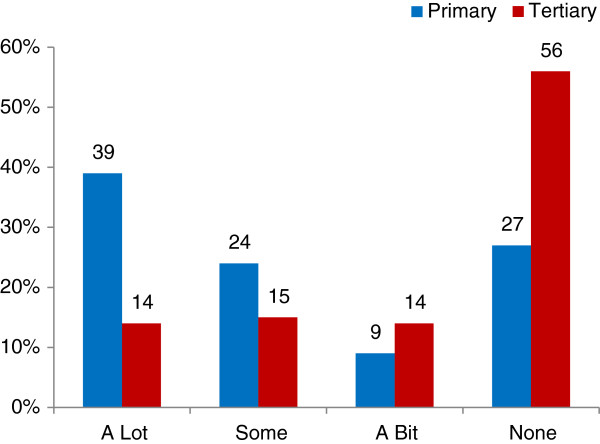
Influence of ‘my general or early pregnancy health’ (survey).

Responses to this question revealed significant differences between the groups, with ‘closeness to home’ (Figure 3), ‘ease of getting there’ (Figure 4), ‘other women’s experiences’, ‘the atmosphere of the unit’ and their ‘own health’ (Figure 5) strongly influential for the PMU group, but not for the TMH group. The strongest factors influencing the TMH group were the ‘availability of specialist services’, with ‘confidence in the staff’ also important (see Table 3). The groups were similar regarding their belief in the minimal influence of both ‘local media’ and ‘the internet’. The combination of ‘none’ and ‘a bit’ included 84% of PMU and 87% of TMH respondents for the local media, and 94% and 98% respectively for the internet.

The groups were equally matched in their survey responses when asked if they agreed with the statement that they ‘were given information about different types of maternity units/hospitals available’, 81% of the PMU group and 80% of the TMH group ‘agreed’ or ‘strongly agreed’, while 11% and 12% of the respective groups ‘disagreed’ or ‘strongly disagreed’. Both groups also concurred that ‘I was able to freely choose the hospital I wanted’, with overall agreement with the statement 94% (PMU) and 88% (TMH) and over two thirds of respondents in both groups ‘strongly agreed’ with the statement. Only 4% (PMU) and 7% (TMH) ‘disagreed’ or ‘strongly disagreed’ with the statement.

In the focus group discussions, both groups identified trade-offs for their chosen birthplace. The TMH women accepted the difficult parking/access, poor communication/support from staff, waiting for care, requirement to transfer immediately after the birth, and even the potential for ‘unnecessary’ intervention (although some believed that they had strategies to avoid it using their midwife as a protective barrier) in order to be ‘safe’. The PMU women accepted the possibility that they may not get to give birth there if complications developed (antenatally or in labour) and the possibility of transfer to the TMH in labour/after birth. They saw the TMH as the place to go only if necessary and the PMU as holistically ‘safer’ for them. In the PMU focus groups there was an awareness of their choice being ‘out of the ordinary’ but their decision was generally well supported by their midwife, partner, family/whanau and at least some friends.

Women who planned PMU births valued accessibility, small size and the atmosphere of the unit - which included the relaxed, homely environment and the care offered by the (midwifery) staff. They liked the flexibility and informality and non-institutional or ‘hospital’ feel of the PMU. For many of the women having previously had a normal birth was influential in their plan to give birth at the PMU for a subsequent birth - it gave them confidence in the process and in their ability to give birth. For these women, having a supportive midwife seemed to facilitate this decision. Having postnatal care experience at a PMU also contributed the decision for some, driven by the desire to avoid the early postnatal transfer and/or to repeat the quality of care and experience in the PMU. While the early postnatal transfer from the TMH is disliked by the participants, it resulted in more women choosing to give birth at a PMU subsequently.

Finally, at a general level both groups appeared to have quite different perspectives on childbirth itself. The following focus group comments illustrate some of the beliefs expressed by participants in the respective groups:

“I had a couple of people going “oh but it’s all just a natural process and it’s all good and you should be all fine”; well actually if you look around the world most of the women die in childbirth, that’s the riskiest thing women do; I wasn’t terribly impressed with that argument” (TMH, Fay).

“I think [TMH]- it’s a hospital, which if you are sick or if you’ve had an accident, that’s great, that’s exactly what you want; but I wasn’t sick, I was having a baby – it’s a perfectly natural process that millions of women all around the world have managed to do without nice shiny hospitals” (PMU, Ivy).

## Discussion

In contexts where women genuinely have birthplace choices, their decision-making appears to reflect their worldview and personal beliefs, which are strongly influenced by the socio-political and cultural context in which they live. Patterson found women’s birthplace planning “was a complex decision… influenced by their personal, social and cultural history” [17]. In the current study different views and beliefs about childbirth were illustrated by the divergent rationales given by the two groups of women. The TMH women actively and almost exclusively chose it for its specialist services/facilities, in common with previous research [11,13,14]. The availability of pain relief and avoidance of intrapartum transfer was only occasionally mentioned. In contrast, the PMU women often gave several reasons, with closeness to home, ease of access, avoidance of early postnatal transfer, the atmosphere or feel of the unit most frequently mentioned. Avoidance of ‘unnecessary intervention’ was also important for some. Previous research also found most of these factors to be important [6,7,10]. Early postnatal transfer from the TMH to a PMU for a couple of days postnatal care may be a context specific factor influencing birthplace decision-making, as it is not discussed in literature from other contexts. In the present study there was congruence between the survey and focus group responses within each group, regarding the reasons the women gave for their birthplace choice, and their relative importance.

Almost all of the respondents appeared to have consciously and actively chosen their birthplace, and identified themselves as the most influential birthplace decision-maker. They reported that their partners had some influence along with some of their midwives, but family and friends had limited influence and doctors had almost none. Overall they agreed that they were given information about different types of maternity units/hospitals and had a free and informed birthplace choice. The PMU group were more likely to be influenced by their midwife. This is understandable in this context where opting to give birth at a PMU is effectively countercultural, given the predominance of the medical model of childbirth and the beliefs associated with it; such as the focus on ‘risk’ and the perception of birth as unpredictable and only ‘normal in retrospect’, the belief that hospital is a ‘safety guarantee’ and that technology does no harm (Table 1) [2,24,25,45].

The influence of the ‘medical or technocratic model’ is evident in the reasons and rationale given by TMH women in the survey and focus groups, in common with previous research [9,16]. They were committed to giving birth there regardless of any other factors- they wanted to be where the specialist services and facilities were ‘just in case’ they were needed, however unlikely this was. The TMH focus group participants expressed a belief that the TMH was the only ‘safe’ place to give birth; arguably for everyone, but certainly for themselves. Some accepted the subjugation of other aspects of ‘safety’ as part of the being at the TMH, a finding in common with Gallagher [Gallagher unpublished observations]. Some acknowledged the increased risk of intervention, but believed that it was a risk worth taking as they saw not being there as more risky for them and/or their baby. They did not perceive that there was any risk of greater morbidity for them or their baby by going to the TMH. For them birth is a ‘high risk’ event and they believe that technology and obstetrics can eliminate, or at least mitigate this risk regardless of personal consequence. The focus groups also revealed an opinion held by some of the TMH focus group participants that women planning PMU births rejected all modern technological advances and would risk their own and/or baby’s wellbeing for ‘a nice sort of birth’, such as the calm relaxed environment or being close to home. Interestingly, this perception was not articulated in the PMU groups, with no PMU woman indicating a rejection of technology or specialist facilities - if they were needed. Overall, the TMH women expressed confidence in their midwives, the specialist services and the system, but not in their ability to give birth and/or the process of birth itself.

In contrast, the influence of the ‘midwifery or holistic model’ is evident in the reasons and rationale given by PMU women, as previously reported ( [6,7,16], Gallagher unpublished observations). The PMU women expressed a belief that the PMU was the best place for them to give birth, although for them it was due to multiple factors which combined to make it feel right. Patterson also found that women sought “a space that felt right and safe for them” [17], p149. The PMU group expressed a belief in it being a ‘safe’ choice, although changeable if any complications arose. Women’s sense of ‘safety’ appears to be central to their decision-making; although the two groups used different means to achieve the same goal, as identified by others previously [1,7,46-48]. The PMU women accounted for physical safety within the established boundaries for being admitted to or transferred out of the PMU, so could then consider their emotional and psychological safety and meeting their personal/social needs. Again, this was in common with Gallagher’s Masters research, which found that the PMU women believed that “they would be in the right place at the right time” [Gallagher unpublished observations p169]. The PMU women expressed confidence in their ability to give birth and in the process of birth. They also had confidence in their midwives and the system of referral and response, if needed.

It appears that the TMH women hold to the core tenets of the ’medical’ model of childbirth and the PMU women reflect the thinking of the ‘midwifery’ model, despite all of the women living in the same socio-political and cultural context. These different perspectives are significant - by identifying with the medical model women do not have confidence in the birth process and believe that they need to have technology and medical specialists for a ‘safe’ birth. (Recent evidence indicates no greater safety is accorded to them, with comparable clinical outcomes for well women giving birth at PMUs when compared with TMHs PMU [27,29,30].) While the two models of birth may be better represented on a continuum than as two separate (and opposing) world views [26], overall the participants in this study expressed two distinctly different sets of beliefs and values on childbirth. The models represent different ways of perceiving and defining the complex concepts of risk, safety, choice and control, which is beyond the scope of this article, but will be addressed in a subsequent article. We argue that these different sets of beliefs strongly influence women’s birthplace decision-making.

This is the first study which has compared birthplace decision-making in the context of universal midwifery-led continuity of care (see [20]). Previous research has identified the opportunity to have ‘a known midwife’ providing labour/birth care as a reason for women to choose a PMU [6,7,10]. Continuity of midwifery care however, was provided to all of the EMU study participants, regardless of their intended birthplace. Arguably, continuity of care may have facilitated women to see themselves as active decision makers, though for many the decisions they make remain strongly influenced by the dominant medical ideology. It is unclear if having their own midwife resulted in women making different birthplace choices from those in other contexts, as New Zealand has a history of a greater proportion of women giving birth in PMUs than in similar resource-rich Western countries. Clearly, if women are to choose a PMU for their birth there first needs to be a PMU in the vicinity. Some women in this study chose their midwife to fit their birthplace choice, while some had limited choice of midwife. Some midwives encouraged women to go to either a PMU or the TMH in line with their personal practice preference. The women’s responses indicated that some midwives were oriented towards the ‘social’ model while others were oriented towards the ‘medical’ model of birth. Overall the women expressed confidence in ‘their’ midwives, the continuity of care they provided and the maternity system. However, this confidence was not enough to over-ride the predominant socio-cultural belief in hospital as the ‘right’ place for the TMH women.

### Limitations and strengths

The New Zealand arm of the EMU study was compromised by damaging earthquakes to Canterbury which started in September 2010 and resulted in the premature end of recruitment, some disruption to birthplace choices and generalised stress and trauma for the whole community. The most severe earthquake in February 2011 killed 182 people and injured many hundreds. More than fifty thousand buildings were seriously damaged, along with waste water and road networks, and the city’s power and water supply were interrupted for some time. Two of the primary maternity units closed for a short period of time, and one was damaged beyond repair and later demolished. The physical impact of the earthquakes varied throughout the city, with the land and buildings in some areas damaged beyond repair and others largely unscathed. The quakes’ effect on residents’ emotional and psychological wellbeing is more difficult to measure, with some seriously traumatised by them at the time and others less so.

It is not possible to quantify the quakes’ impact on individual participants or identify if one cohort was more adversely affected that the other. No differences were identified in the focus groups and no women gave ‘earthquakes’ as a reason for their initial birthplace choice in the survey, despite approximately 40% of participants joining after the first earthquake. The earthquakes did force some changes to birthplace plans for study participants, and these will be addressed in a subsequent article focusing on antenatal changes of plan and transfers. Of note is that throughout the quakes most participants returned the study surveys, and most of those who had indicated their interest in the focus group prior to the quakes attended groups, when given the opportunity a whole year later. This suggests that despite the stress and trauma around them they wanted to share their experiences or ‘tell their story’ for the EMU study.

The halt to recruitment resulted in a smaller sample than planned, with more women in the PMU group, due to the initial protocol of not making follow-up calls to those booked into the TMH for the first six months of recruitment.

While it was intended to explore issues of cultural safety and differences for Māori women in the EMU study, which included a focus group run by Māori for Māori. As a result of the earthquake related disruption the focus group was not undertaken. The small proportion of Māori participants (5.6% in the PMU group and only 2.6% in the TMH group) prevented analysis of the survey results by ethnicity.

The sample was biased towards those with a moderate ability to read and write in English, required in order to read the study information and consent forms. Although an interpreter was offered no one took up the offer. Fewer Māori women joined than in the background population. The surveys and focus groups which asked about birthplace planning were undertaken postnatally, potentially influencing responses. Self selection bias is present in both groups, as all of the women chose their preferred birthplace, so any psychological or motivational differences between the groups cannot be accounted for.

Although smaller than planned, the size of the study is one of its strengths, along with the high survey response rate (571 respondents). Undertaking both survey and focus groups facilitated data comparison, which proved confirmatory and complementary. The survey provided breadth of data and focus groups provided depth on some issues, enabling consideration of the complexity of the issue. The thorough process of focus group transcript and data triangulation ensured robust qualitative data analysis.

### Implications for practice

Research evidence affirming the ‘clinical safety’ of free-standing primary level midwifery-led maternity units addresses only one aspect of the beliefs influencing childbearing women’s birthplace decision-making. In order for them to feel ‘safe’ going to a primary unit, other aspects of women’s beliefs and the complexity of the concepts of ‘risk’ and ‘safety’ need to be identified and explored, and much wider socio-political change will be required. This study provides some guidance for midwives by providing background information as to the reasons women identify for their choice to go to either a primary unit or tertiary hospital, and may inform their birthplace discussions with women and their partners or family/whanau. It can also provide maternity service planners insight regarding the issue of birthplace, which is a profoundly important but generally under-acknowledged part of the journey of childbirth.

## Conclusion

This is the first published study reporting women’s perspectives on their birthplace decision-making in New Zealand. It is also the first study to report this aspect of choice in childbirth where continuity of midwifery care is available to women, regardless of their intended or actual birthplace. It identified that almost all the participants perceive themselves as the principal birthplace decision-maker, with midwives being the most influential health professional. The two groups value different factors, with the tertiary hospital group citing the desire to have specialist facilities available ‘just in case’ as the primary factor, and the primary unit group identifying several factors, including closeness to home, ease of access, the atmosphere or feel of the unit and avoidance of ‘unnecessary intervention’. The groups’ responses expressed different ideological positions which expressed different beliefs about the complex constructs of ‘risk’ and ‘safety’. All of the women in the study were well at the time they joined the study, with no clinical indication for obstetric or medical intervention in birth. Both groups believed their chosen birthplace was the right and ‘safe’ place for them to plan to have their baby.

## Abbreviations

EMU: Evaluating Maternity Units; TMH: Obstetric-led Tertiary level Maternity Hospital; PMU: Free-standing midwifery-led Primary level Maternity Unit; LMC: Lead maternity carer; GP: General Practitioner (family doctor); Quan: Quantitative; Qual: Qualitative; MMPO: Midwifery and Maternity Provider Organisation; NZCOM: New Zealand College of Midwives; COSMOS: Australian randomised controlled trial of caseload midwifery for low risk women [38]; M@NGO: Randomised controlled trial of caseload midwifery [42]; FG: Focus group quotes (pseudonym identifier); S: Survey quotes (‘study code’ identifier).

## Competing interests

All authors declare that they have no competing interests.

## Authors’ contributions

CG led the New Zealand arm of the study and prepared and modified the manuscript. SKT conceived of the study, led its design and coordination. CG, SKT, RD and MK participated in survey design and analysis. CG, RD & MK participated in focus group design, conduct and analysis. All authors (CG, SKT, RD, MK & VS) discussed core ideas, provided critical review and approved the final manuscript.

## Authors’ information

CG – MMid BMid BA, PhD candidate University of Sydney, Australia, RM New Zealand.

SKT – DMID MA RGON RM, Professor of midwifery, the University of Sydney, NSW, Australia.

RD – PhD, Senior lecturer, School of Midwifery, Christchurch Polytechnic Institute of Technology, Christchurch, New Zealand.

MK – MPH BA DipTchg (Tertiary) RGON RM ADM, Co-Head of Midwifery, Principal lecturer, School of Midwifery, Christchurch Polytechnic Institute of Technology, Christchurch, New Zealand.

VS – PhD RM RN, Professor in maternal and infant health, School of Nursing and Midwifery, Family and Community Health Research Group, University of Western Sydney, NSW, Australia.

## Pre-publication history

The pre-publication history for this paper can be accessed here:

http://www.biomedcentral.com/1471-2393/14/210/prepub
